# Cervical Cancer Prevention in the Era of the COVID-19 Pandemic

**DOI:** 10.3390/medicina58060732

**Published:** 2022-05-29

**Authors:** Patryk Poniewierza, Grzegorz Panek

**Affiliations:** 1 Medicover Sp. zo.o, Aleje Jerozolimskie 96, 00-807 Warsaw, Poland; 2 Department of Oncologic Gynecology and Obstetrics, The Center of Postgraduate Medical Education, 00-416 Warsaw, Poland; gmpanek@wp.pl

**Keywords:** cervical cancer, prevention, screening, vaccination, COVID-19

## Abstract

*Background and Objectives*: Cervical cancer (CC) is the fourth most common cause of cancer-related morbidity and mortality among women worldwide. CC prevention is based on screening and HPV vaccination. The COVID-19 pandemic has caused difficulties in implementing CC-preventative measures. The aim of this study was to collect data on the implementation of CC prophylaxis in Poland provided by public and private health care with a particular focus on the impact of the COVID-19 pandemic and attempt to estimate the level of CC-screening implementation by 2026 under public and private health care. *Materials and Methods*: Data on the implementation of privately funded (2016–2021) and publicly funded (2014–2021) CC-preventative measures in Poland were examined. The Prophet algorithm, which positions itself as an automatic forecasting procedure and represents a local Bayesian structural time-series model, was used to predict data. The correlation test statistic was based on Pearson’s product moment correlation coefficient and follows a t distribution. An asymptotic confidence interval was given based on Fisher’s Z transform. *Results*: In 2021, a significantly higher population screening coverage was observed in private health care (71.91%) than in the public system (12.6%). Our estimation assumes that the adverse downward trend of population coverage (pap smear CC screening) in the public system will continue to 5.02% and in the private health system to 67.92% in 2026. Correlation analysis showed that with the increase in the sum of HPV tests and LBC, the percentage of Pap smear coverage in the private healthcare sector decreases r = −0.62, *p* = 0.260 df = 3, CI = [−0.97, 0.57]. The amount of HPV vaccinations provided in private health care is steadily increasing. Immunization coverage of the population of girls aged 9–18 years under private health care at the end of the observation period was 4.3% (2021). *Conclusions*: It is necessary to reorganize the public CC-screening system in Poland based on a uniform reporting system for tests performed in both public and private health care using the model of action proposed by us. We recommend the introduction of a national free HPV vaccination program funded by the government and implemented in public and private health care facilities.

## 1. Introduction

In 2020, cervical cancer (CC) was the fourth most common cancer (604,000 new cases), resulting in 342,000 deaths in women worldwide. In Europe, 58,169 new cases were diagnosed (9.6% of worldwide diagnoses), with 25,989 deaths (7.6% worldwide) [1,2]. Poland ranks eighth in the European Union (EU) in the incidence of CC and fifth in deaths from this disease. The worldwide 5-year survival rate for CC is 48.3% versus 62.1% for EU countries [3].

Analysis of the trend over several decades indicates a systematic decrease in both morbidity and mortality due to CC in most countries, including Poland [4,5]. This phenomenon has been attributed to improved public awareness and better economic conditions [6,7]. These improvements have translated into fewer infections with the high-risk human papillomavirus (hrHPV) strains (i.e., HPV 16, HPV 18) responsible for the onset of CC and the widespread availability of preventative methods, such as screening and vaccination [8]. Screening for CC is based on the use of cervical cytology, which can be done with a Pap smear or liquid-based cytology (LBC), or via more sensitive HPV DNA testing [9,10]. A currently postulated extension of the above approaches is double immunocytochemical staining in cervical cytology samples for the antiproliferative proteins p16 and Ki67 [11,12]. 

HPV infections are transmitted sexually, and most are transient and produce no symptoms. However, hrHPV infections, due to their chronic nature, can lead to the development of cervical, anal, penile, and throat cancers depending on the route of sexual activity [13,14]. The primary prevention of CC involves vaccination against HPV types 6, 11, 16, and 18 (Gardasil/Silgard); types 16 and 18 (Cervarix); and types 6, 11, 16, 18, 31, 33, 45, 52, and 58 (Gardasil 9). HPV vaccination is available for people aged 9 and older, and it is recommended that the vaccination be given before sexual activity begins. By 2020, 200 million doses of HPV vaccination had been administered worldwide [15,16]. 

Individual countries around the world have used the methods listed above (CC screening, HPV vaccination) to create national prevention programs. In Poland, an organized screening program for CC was established in 2006, which involves performing Pap smear-based cervical cytology on women aged 25–59 years at 3-year intervals [17]. In December 2021, The Polish Society of Gynecologist and Obstetricians published recommendations including liquid-based cytology (LBC) in the regimen for cervical cancer (CC) screening. However, it remains outside the national screening program and is available for a fee through private health care [18]. The 20 percent population coverage of CC screening under public health care in Poland, with declining mortality rates, suggests that many preventive screenings are performed under private health care. However, the exact numbers are not known—it is estimated that this number may be as high as two-thirds of all CC-screening tests performed [19,20].

Currently, the National Cancer Institute in Poland is piloting the use of HPV testing in women aged 30–59 years [21]. At present, Poland does not have a national HPV vaccination system, and these initiatives are implemented locally from the budgets of municipalities or cities. Other EU countries that do not have a national HPV vaccination program include Bulgaria, Romania, and Slovakia [22,23,24,25]. 

In 2020, the World Health Organization (WHO) unveiled its global strategy to accelerate the elimination of CC as a public health problem, which aims to achieve a level of four cases per 100,000 women-years for this disease. The keys to achieving this goal by 2030 include an HPV vaccination rate of 90% for girls up to 15 years of age, screening 70% of women aged 35 to 45 years at least twice and assuring that 90% of women diagnosed with precancerous lesions or CC receive adequate treatment [26,27].

The implementation of this strategy has become a significant challenge in the era of the coronavirus disease 2019 (COVID-19) pandemic. Impeded access to health care services, supply chain disruptions, and deteriorations in the financial health of countries are all factors that have undermined the implementation of CC-prevention measures [28,29,30,31].

The aim of the current study was to examine the implementation of CC-preventative measures in the public and private health care sectors in Poland, with a special focus on the impact of the COVID-19 pandemic. It is the intention of the authors that, based on the Polish experience, the correlations detected, and solutions proposed can become a starting point for the creation of preventative solutions with a greater effectiveness and resistance to external factors (such as epidemics or disasters) that will enable the achievement of the goal set by the WHO.

## 2. Materials and Methods

### 2.1. Study Design

The purpose of our study was:

- To collect and compare data on the implementation of CC prevention in Poland under the public health care from the resources of the National Health Fund (NHF), which is the only entity in Poland who finances public health services from obligatory health insurance fees, and in the private sector on the example of one of the main private medical service providers in Poland (subscription-paid system);
- To consider the impact of the onset of the COVID-19 pandemic on the level of implementation of CC prevention in public and private health care;
- An attempt to estimate the level of CC-screening implementation by 2026 under public and private health care;
- To propose implementation schemes for CC prevention resilient to external factors such as pandemic, disaster, or war.

The period analyzed is 2014–2021 for public health care and 2016–2021 for private health care. The differences in the study period are due to missing data on the private provider side. Patients covered by private a health care will be referred to as “members” later in this study.

The principles of CC prevention in public health care in Poland are presented in Table 1 [5,17,18,22,24], and information regarding the private health care based on the example of the provider included in our study are presented in Table 2 [32].

### 2.2. Data

#### 2.2.1. Public Health Care

Our study used data made available by the NHF at the request of the authors.

##### Screening CC

Data on the number of cytology examinations performed refer to all cervical cytology examinations (Pap smear) performed between 2014 and 2021, which were funded by the NHF under the CC-screening program. According to the NHF’s methodology, the percentage of population coverage for screening refers to the number of women screened with a Pap smear of the cervix in a given year versus the number of women eligible for screening in a given year. LBC and HPV testing are not covered by the program.

#### 2.2.2. Private Health Care

Data provided by health care provider upon authors’ request.

##### Screening CC

In this part of the study, we used data for the period 2017–2021 relating to screening tests performed (Pap smear) as part of the ongoing screening program. As with public health care, only performing a Pap smear on female members is used to report the percentage of screening coverage. Other screening methods (LBC, HPV test) are available. However, they are not included in the reporting of the percentage of screening coverage, as they are available upon a fee.

##### HPV Vaccination

HPV vaccination data from 2016 to 2021 refer to the number of doses of vaccination administered using a medically qualified formulation held by a health care provider. Prescriptions with the option to purchase the product at a pharmacy and vaccinate at another medical facility are not dispensed. Ongoing monitoring of population coverage refers to female members aged 9–18 years who have received a full course of HPV vaccination by the end of 2021.

##### Preventative Actions

The prevention campaigns described in the study are training sessions for members covering CC prevention with their topics, which took place in 2019–2020 (on site) or as webinar (online) format in 2021.

#### 2.2.3. Other

We also used demographic data from the Polish population that are published by the Central Statistical Office and information on the functioning of the state telemedicine platform P1 [33,34]. 

### 2.3. Statistical Analyses

Analyses were conducted using the R Statistical language (R Core Team, Vienna, Austria version 4.1.1) on Windows 10 PRO 64 (build 19044), using the packages Rcpp (https://www.jstatsoft.org/v40/i08/, version 1.0.7, accessed on 30 April 2022), ggplot2 (New York, NY, USA, version 3.3.5), forecast (https://pkg.robjhyndman.com/forecast, version 8.15, accessed on 30 April 2022), rlang (https://CRAN.R-project.org/package=rlang, version 1.0.1, accessed on 30 April 2022), report (https://github.com/easystats/report, version 0.5.1, accessed on 30 April 2022), and Prophet (https://CRAN.R-project.org/package=prophet, version 1.0, accessed on 30 April 2022) [35,36,37,38,39,40,41].

#### 2.3.1. Prediction

The Prophet algorithm, which positions itself as an automatic forecasting procedure and represents a local Bayesian structural time-series model, was used to predict data.

The Prophet algorithm was an appropriate tool for the analysis of studied data because it is based on an additive model that fits nonlinear trends, including data with an annual granularity. It works best with multiple seasons of historical data and generally handles outliers well also with non-stationary data [42,43]. 

The Prophet algorithm’s principle is based on a three-component model (trend, seasonality, and holidays) decomposition of time-series data using estimation procedures for structural time-series models [44].

*Y*(*t*) = *g*(*t*) + *s*(*t*) + *h*(*t*) + *ϵ_t_*, (1)

The trend function *g*(*t*) was used to model non-periodic changes in the time series’ value, *s(t)* was used to model periodic changes (e.g., annual seasonality), and *h*(*t*) was used to model the effects of holidays that occur at potentially irregular intervals over one or more days. The error term *ϵ*t corresponded for any idiosyncratic changes that the model did not account for. For the trend model, the linear one was chosen with the following equation:

*g*(*t*) = (*k* + *a*(*t*)*^T^ δ_j_*)*t* + (*m* + *a*(*t*)*^T^ γ_j_*), (2)

where *k* was the growth rate, the rate adjustments that occur at time *sj* are represented by *δj*, and *a*(*t*)—vector of adjustments (∈ {0;1}^S^). To make the function continuous, *m* (the offset parameter) was set to −*s_j_δ_j_* (*s_j_* was the changepoint times *j* = 1, …, S), and *j* was set to −*sjj* (*sj* were the changepoint times *j* = 1, …, S).

The uncertainty of the predicted trend was estimated by extending the generative model forward. The generative model for the trend implied that there were S changepoints over a history of T points, each of which has a rate change *δj*~Laplace(0, τ). The τ parameter directly controlled the model’s flexibility when modifying its rate. Simulation of future rate changes mimicking those of the past was achieved by replacing τ with a variance derived from the data. It was done by prior application of a Bayesian framework with a hierarchical for τ to obtain its posterior; otherwise, the maximum likelihood estimate of the rate scale parameter, namely $\lambda =\frac{1}{S}\overset{S}{\underset{j=1}{\sum }}\left|\delta_{j}\right|$, was applied. Modeling seasonality relied on the standard Fourier series to provide a flexible model of periodic effects [45].

(3) $$s\left(t\right)=\overset{N}{\underset{n=1}{\sum }}\left(a_{n}cos\left(\frac{2\pi nt}{P}\right)+b_{n}sin\left(\frac{2\pi nt}{P}\right)\right),$$

The seasonality adjustment *s* was achieved by constructing a matrix of seasonality vectors for each value of *t* of historical and future data. The entire model from (1) for current project in Stan code is shown as follows [46]:

(Prior for Holidays and events, component *h*(*t*) was not reported because it is not applicable in current research).

Model {m ∼ normal(0, 5); // the offset parameter epsilon ∼ normal(0, 0.5); delta ∼ double_exponential(0, tau); //automatic changepoint selection beta ∼ normal(0, sigma); //seasonality y ∼ normal((k + A * delta). * t + (m + A * gamma) + X * beta, sigma); //Linear likelihood}.

For Stan’s model fitting the limited memory, Broyden–Fletcher–Goldfarb–Shanno algorithm was implemented to find a maximum of a posterior estimate [47].

The Prophet forecaster stage of the current project was applied with linear growth, with the trend uncertainty using the maximum a posteriori (MAP) estimate of the extrapolated generative model (0.8) and the flexibility of the automatic changepoint selection (0.95). 

Data predictions were made based on the data from period of 5 years (from 2017 to 2021) in the case of private health care and of 8 years (from 2014 to 2021) in the case of public health care. The prediction period was set at 5 years (from 2022 to 2026). The threshold for determining the significance of the absolute value of the delta change points was set at the level of 0.1. 

#### 2.3.2. Correlation

The correlation test statistic was based on Pearson’s product moment correlation coefficient and follows a *t* distribution with length (pairs of observation—2) degrees of freedom. An asymptotic confidence interval was given based on Fisher’s Z transform.

### 2.4. Ethics

The study we present is not a medical experiment and does not require approval from a bioethics committee. 

## 3. Results

### 3.1. Cervical Cancer Screening

The data (Table 3) show a 17% decrease in the number of women screened at publicly funded facilities in 2020 compared to 2019. This decrease is associated with the beginning of the COVID-19 pandemic in Poland, when restrictions on access to health care were introduced during the first wave of the pandemic. Compared to 2014, population coverage for CC screening declined by a total of 9.74 percentage points in 2021—a clear downward trend. Over the entire observation period, the largest year-to-year decrease was observed in the first year of the pandemic in Poland (2.38 percentage points). 

As the private sector did not limit access to medical services during the pandemic, this resulted in an only 1% decrease in the number of women screened in 2020 relative to 2019 (Table 4). Despite the observed decline, population screening coverage in 2021 was significantly higher in private health care (71.91%) than in the public system (12.6%).

Despite the increasing number of women screend in private health care, 143,077 in 2021 compared to 106,885 in 2017, a decrease in the percentage of population coverage is observed. In objectively assessing the merits of the private health care model, we assumed that the decline observed was not due to hypothetical inefficiencies in the private model but to the rapid growth in the number of people using the private sector as well as the use of other screening methods available within the system and not counted in the reporting (Table 5).

The correlation analysis showed that as the number of members increased, the percentage of Pap smear screening coverage for private health care significantly decreased (Pearson correlation coefficient r = −0.89, *p* = 0.046, degrees of freedom df = 3, confidence interval CI = [−0.99, −0.01]), and the correlation analysis also showed that despite the increase in the sum of HPV testing and LBC, the percentage of Pap smear screening coverage in the private health care sector decreased (r = −0.62, *p* = 0.260 df = 3, CI = [−0.97, 0.57]).

This indicates that as the number of people increases, the percentage of coverage decreases, and as the performance of other screening tests increases, the percentage of population coverage strongly decreases. These results confirmed our assumptions and provided a recommendation to revise the current reporting rules.

We attempted to estimate population coverage by 2026 for publicly and privately funded cervical cancer screening by assuming no change in the current approach to the screening programs.

The Prophet algorithm (public health care CC screening) allowed for a decent fit to the data (the median absolute percentage error (MdAPE) = 0.04, and symmetric mean absolute percentage error (SMAPE) = 0.06). 

**Figure 1:**
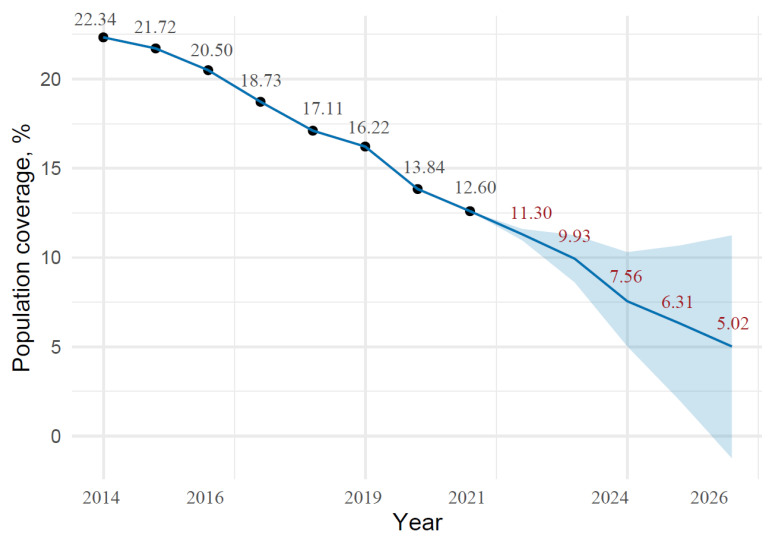
Population coverage and projection of CC screening in public health care from 2014 to 2021, with Prophet algorithm predictions for 2022–2026.

A time-series plot of the data along with the forecast data is shown in Figure 1.

The plot shows a decrease in the percentage of the population covered in the public care sector from 12.60% at the end of 2021 to 5.02% in 2026. Projection results along with confidence interval levels were presented in Table 6

The Prophet algorithm (private health care CC screening) allowed for a decent fit to the data (MdAPE = 0.09 and SMAPE = 0.34).

**Figure 2:**
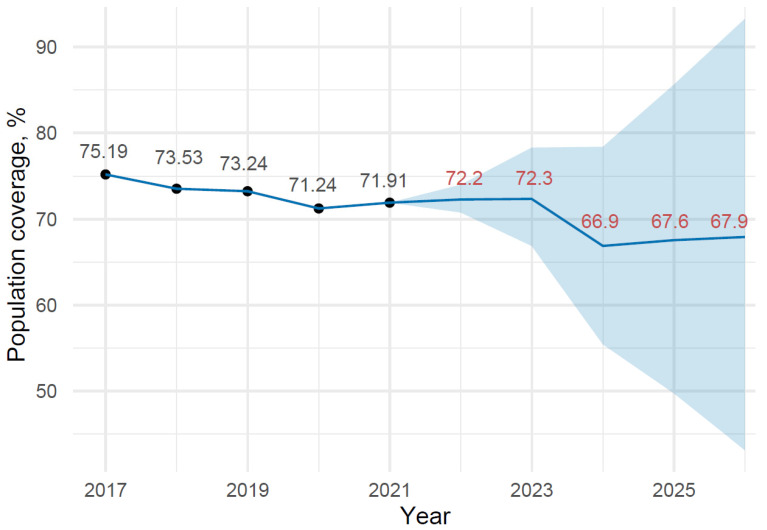
Population coverage and projection of CC screening in private health care from 2014 to 2021, with Prophet algorithm predictions for 2022–2026.

A time-series plot of the data along with the forecast data is shown in Figure 2.

The plot shows a decrease in the percentage of the population covered by CC screening in the private care sector from 71.91% at the end of 2021 to 67.9% in 2026. Projection results along with confidence interval levels are presented in Table 7.

The data we present here relate only to one private health care provider in Poland. The lack of a common database for private and public health care providers and uniform reporting criteria makes it impossible to conclude on the real coverage for the entire Polish population for CC screening.

### 3.2. Human Papillomavirus Vaccination

**Figure 3:**
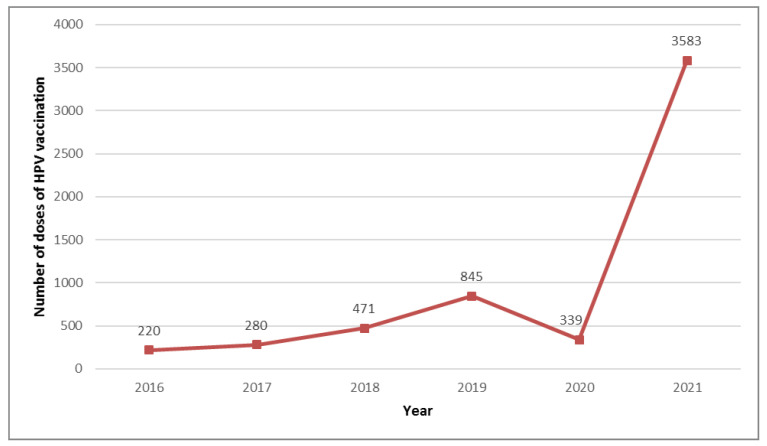
Number of doses of HPV vaccination administered (private health care).

There is no publicly funded national HPV vaccination program in Poland. The number of vaccination doses administered in private health care is shown in Figure 3.

There is a systematic upward trend despite a decrease in the number of doses administered in the first year of the pandemic (2020). Immunization coverage of female members (aged 9–18 years) at the end of the observation period was 4.3% (2021) as shown in Table 8.

We also analyzed the interest of patients covered by private medical care in preventative actions and thematic meetings (on site or online), including HPV vaccination and CC screening (Table 9).

In the first year of the COVID-19 pandemic, there was a decline in interest in this topic. However, 2021 has already seen an increase of more than 2.5 times that of 2019. Contributing to this was the improved accessibility to training, as face-to-face meetings were changed to webinars. 

The data presented here do not distinguish between patients in terms of urban or rural residence. However, the planned organization of HPV vaccination should pay special attention to the problem of rural areas, as they are inhabited by 40% of the Polish population.

## 4. Discussion

### 4.1. Cervical Cancer Screening

The observed decline in the number of women screened in the public health system in 2020 in Poland that was associated with the COVID-19 pandemic is in line with the trends observed in other countries. According to estimates, disruptions in screening worldwide could increase CC cases by an average of 5–6% [48]. The delay in screening is not insignificant in terms of its impact on treatment outcomes. Brazilian data show that the percentage of women with FIGO stage III–IVa diagnoses increased from 43.3% before the pandemic to 56.8% during the pandemic [49]. 

The magnitude of the decline in screening in different countries varies and depends on the organization of national prevention programs (HPV DNA tests or Pap smears). Countries such as Poland, which have a Pap smear-based screening program, have proven to be the most vulnerable to the pandemic [50,51]. Australia, which introduced HPV testing every 5 years in 2017 instead of the previously used cervical cytology performed every 2 years, was only affected by the pandemic in a minor way. More than 90% of screened women were found to be HPV-free, allowing the next HPV test to be deferred until the end of 2022 [52]. The cited strategy is a recommendation for the revision of standards of conduct in other countries of the world, including Poland. Another noteworthy initiative is HPV self-sampling. This method can be used in times of impeded access to health care services (e.g., the COVID-19 pandemic) as well as on a permanent basis for countries with low CC screening or locally in areas with limited medical resources (e.g., rural areas). Of the 48 countries worldwide with programs based on HPV testing, 35% have already updated their guidelines to recommend self-sampling. A Dutch study of 180,000 women showed that results achieved by self-sampling and by physician testing do not differ in sensitivity or specificity [53,54].

Policy decisions to suspend CC screening were intended to protect both screening participants and medical personnel from potential SARS-CoV-2 infection. After the first wave of the pandemic subsided, individual countries made attempts to mobilize and make up for the resulting shortfalls [55]; however, we must wait to assess the effects of these efforts. Without being able to rule out further pandemics or natural disasters negatively affecting the implementation of prevention programs in different regions of the world [56], we consider it necessary to introduce a fixed-parameterized scheme of action for the return to preventative testing consistent with our recommendations (Table 10).

In addition to the obvious decline in screening due to its deferral by healthcare providers during the first wave of the pandemic, the decline in interest in preventative screening among patients itself is not insignificant [57]. A 2020 study examining the number of Google searches for the phrase “pap smear” showed that there was 54.1% decrease in the use of these search terms compared the same period before the pandemic [58]. Our results from the private sector, which did not limit access to the medical services provided during the pandemic, demonstrate a very unique approach to service delivery as well as a patient perspective not previously reported in the literature. 

The results of our study indicate a decrease in the percentage of population coverage for CC screening over the follow-up periods in both the public and private systems. However, the reasons for this phenomenon vary. Underlying the declines for private health care are reporting errors (failure to include other screening methods in the data) and dynamic membership growth, making it impossible to compare the figures year-over-year. The reasons for the weakness of the public CC-screening system in Poland likely include a lack of personalized invitations to cervical cytology examinations, low social awareness, a lack of modern diagnostic methods to respond to the needs of a more informed population, and a lack of sufficient financing [59,60]. In contrast to this approach is the private medical care system, where patients are regularly educated (preventative campaigns) and proactively encouraged to undergo screening. Not without significance is the availability of a wide range of diagnostic services, including cytology taken by a doctor or midwife and the possibility of performing LBC or HPV DNA tests. A study based on 30,066 screening tests (LBC, hrHPV, p16/Ki67) performed in private outpatient gynecological clinics showed that the private model can be effectively used for CC prevention [12]. The unfavorable prognosis we discovered regarding the implementation of the publicly funded CC-screening program in Poland allows us to propose the following corrective actions presented in Table 11.

The COVID-19 pandemic revealed vulnerabilities in the global supply chain for medicinal products, personal protective equipment, and diagnostic supplies [61]. This undoubtedly has had an impact on the implementation of screening programs. In a group of 57 HPV DNA-testing laboratories from 30 countries, as many as 74% reported a lack of supply as well as staff shortages (54%) [62]. It should be noted that the laboratories that participated in this latter study also carried out SARS-CoV-2 PCR assays, which may have influenced the results due to shifting diagnostic lines. Nonetheless, the direction of inquiry seems appropriate, and it is worth evaluating the impact of the COVID-19 pandemic on the delay in shipping Pap results by cytology laboratories due to equipment shortages and staffing issues.

**Figure 4:**
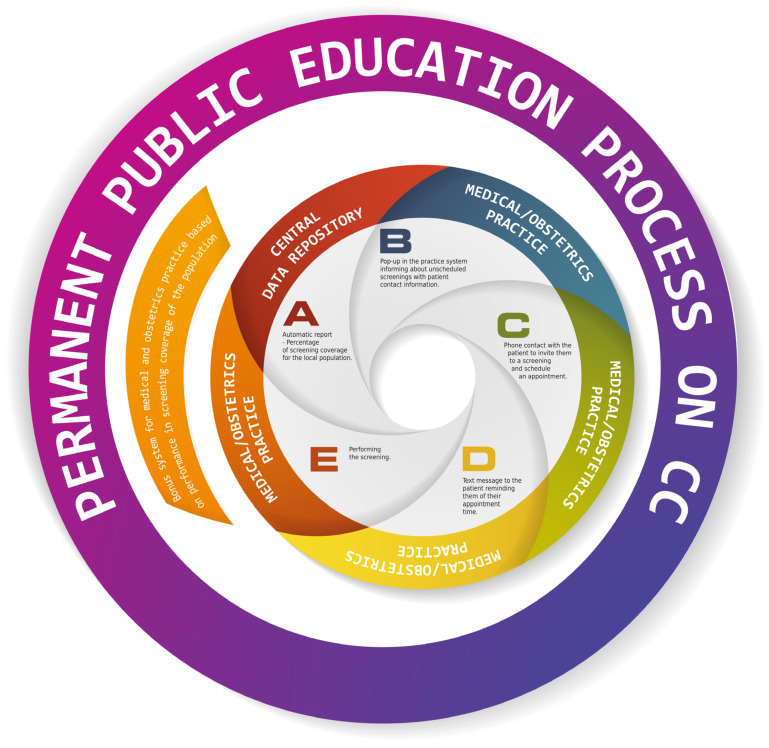
Recommended model for cervical-cancer-screening system operation.

In light of the above arguments, the situation in countries with low levels of CC screening appears to be particularly difficult and was further exacerbated by the COVID-19 pandemic. In Poland, the population coverage rate for the national CC-screening program was 16.22% in the year prior to the pandemic. Thus, it was significantly lower than the average for 15 other EU countries [63]. As inequalities continue to widen, it makes sense to create effective screening programs for countries that are doing poorly in CC prevention. The IT infrastructure that has been developed by the Ministry of Health in Poland to address the COVID-19 pandemic may be helpful. The implemented telemedicine solutions within the P1 platform for collecting, analyzing, and sharing medical data in digital form can also be used in CC prevention [64]. Our suggested target common model for screening system operation, regardless of funding source (private or public) and based on the existing IT infrastructure in Poland, is presented in Figure 4.

### 4.2. Human Papillomavirus Vaccination

In the era of the COVID-19 pandemic, it may prove extremely difficult to achieve the WHO’s goal of eliminating CC as a global public health problem. It is predicted that this goal can be achieved with 90% HPV vaccination coverage in the general population and 95% in high-risk groups [65]. The level of global coverage in 2019 was estimated to be 15% [66]. As noted in the introduction, Poland does not have a national public HPV vaccination program, and vaccination is carried out locally by local government units. As such, there is no central reporting of the vaccination rates in the Polish general population. The most recent study covering the period from 2009 to 2016 examined 1204 local HPV vaccination programs and reported a 2.05% vaccination rate for girls aged 10–14 years nationwide [67]. This is lower than that reported in private health care and far away from rates necessary for the global goal of eliminating CC. A step in the right direction is the 50% reimbursement for HPV vaccine costs introduced by the Polish government in November 2021; however, it is still too early to assess the effects of this measure [68]. 

The current results also show a steady increase in HPV vaccination rates in private health care, with an apparent decline in 2020. The WHO and UNICEF have both highlighted the decrease in vaccinations given to children due to the COVID-19 pandemic. The annual number of HPV vaccine doses administered decreased by 24% in 2020 compared to 2019. Among the childhood vaccines, this was the largest decrease observed (e.g., Tdap rates decreased by 21.2%, and meningococcal ACWY decreased by 20.8%). The above correlation may be indicative of the “categorization” of vaccine validity that parents have implemented. It is estimated that the global deficits in vaccination rates will not be offset until 2031 [69,70]. However, an analysis of Google searches indicates an unchanged level of interest in HPV vaccination since the beginning of the pandemic compared to the “pre-COVID-19” period [71]. An important observation is that parental awareness of HPV vaccines in Poland is unsatisfactory and requires national remedial action [72]. Based on the results from private health care and the fact that there is no public HPV vaccination program, we propose to take the actions included in Table 12.

Although the data presented in the current study do not distinguish between urban and rural areas, previous studies have indicated that, in populations with high vaccination rates, the percentage of HPV vaccination in rural areas is 10% lower than in urban areas. This is due to several factors, including lower public awareness, a reduced interest in conducting preventative activities by medical personnel, and a limited number of outlets providing medical services [73]. For countries such as Poland, where a significant proportion of the country’s population lives in rural areas, it is critical to develop methods to promote prophylactic HPV vaccination in these regions. 

## 5. Conclusions

In conclusion, we would like to highlight the following findings:

(a) Patient interest in publicly funded CC screening has steadily declined each year. The COVID-19 pandemic significantly exacerbated this adverse trend.
(b) The percentage of population coverage with Pap smear-based screening for both public and private health care is expected to continue to decline over the next few years.
(c) The private CC-screening model has a higher efficiency measured as a percentage of population coverage. There is a growing patient interest in the more modern screening methods, such as LBC and HPV testing. Reporting based solely on Pap smear becomes inadequate.
(d) The approach to the onset of the COVID-19 pandemic in private health care had a smaller effect on the decline of interest in CC screening compared to the effect observed within public health care.
(e) Based on private health care data, the percentage of the Polish population vaccinated against HPV should be considered insufficient.

In view of the WHO’s call for the elimination of CC as a global public health problem and the results of our study, we call for immediate action to improve CC prevention in Poland. It is necessary to reorganize the public CC-screening system in Poland based on a uniform reporting system for tests performed in both public and private health care using the model of action proposed by us. We recommend the introduction of a national free HPV vaccination program funded by the government and implemented in public and private health care facilities.

## Figures and Tables

**Table 1:** The principles of CC prevention in public health care in Poland.

Public Health Care	
Cervical Cancer Screening	HPV Vaccination
A national screening program based on Pap smear Criteria for inclusion of women in the program: age 25–59 years, with cytology (Pap smear) repeated every 3 years. LBC and HPV tests are not covered by the standard public health care Failure to actively invite patients by name to participate in screening	Lack of a population-based HPV vaccination program funded by the state budget

**Table 2:** The principles of CC prevention in private health care in Poland.

Private Health Care	
Cervical Cancer Screening	HPV Vaccination
Pap smear-based screening program Inclusion criteria for women in the program: age 25–65 years, with cytology (Pap smear) repeated every 3 years. LBC and HPV tests available as a paid test outside the standard of care Personalized invitations to the CC-screening examinations	No free HPV vaccination program funded by subscription to the private health care Purchase of the HPV vaccine on a fee-for-service basis; eligibility for vaccination for free within subscription Vaccination is performed with the self-bought vaccine that is in the possession of the medical facility after prior clearance by a doctor (issuance of a referral for vaccination; pharmacy prescription is not issued) Actively inviting patients to get vaccinated

**Table 3:** Population coverage of publicly funded cervical cancer screening as part of a prevention program in Poland.

Publicly Funded Cervical Cancer Screening (Pap Smear)			
Year	Number of Women Screened	Number of Women Qualified for the Screening	% Population Coverage
2014	2,212,647	9,906,366	22.34
2015	2,148,973	9,894,022	21.72
2016	2,028,217	9,896,007	20.5
2017	1,846,369	9,855,788	18.73
2018	1,689,552	9,874,141	17.11
2019	1,614,045	9,953,205	16.22
2020	1,380,428	9,977,646	13.84
2021	1,267,119	10,058,829	12.6

**Table 4:** Population coverage for privately funded cervical cancer screening.

Cervical Cancer Screening (Pap Smear)			
Year	Number of Women Screened	Number of Women Qualified for the Screening	% Population Coverage
2017	106,885	142,157	75.19%
2018	116,485	158,419	73.53%
2019	128,588	175,578	73.24%
2020	127,308	178,711	71.24%
2021	143,077	198,967	71.91%

**Table 5:** Number of tests performed (LBC and HPV tests) and number of members—private health care.

	Criterion	LBC	HPV Tests	Total Number of Members Covered by Private Health Care
Year				
2017		0	597	495,242
2018		0	824	559,246
2019		8	985	620,595
2020		57	877	655,649
2021		107	1669	728,031

**Table 6:** Projection of population coverage for publicly funded cervical cancer screening as part of a prevention program in Poland.

Year	$\hat{y}$	*CI_ll_*	*CI_ul_*
2022	11.30	10.97	11.60
2023	9.93	8.61	11.25
2024	7.56	5.03	10.30
2025	6.31	2.00	10.67
2026	5.02	<0.00	11.24

$\hat{y}$, the predicted value; *CI_ll_*, lower level of confidence interval; *CI_ul_*, upper level of confidence interval.

**Table 7:** Projection of population coverage of privately funded CC screening in Poland.

Year	$\hat{y}$	*CI_ll_*	*CI_ul_*
2022	72.28%	70.49%	73.78%
2023	72.35%	65.37%	77.85%
2024	66.88%	53.69%	77.81%
2025	67.55%	46.37%	84.80%
2026	67.92%	38.10%	93.18%

$\hat{y}$, the predicted value; *CI_ll_*, lower level of confidence interval; *CI_ul_*, upper level of confidence interval.

**Table 8:** Percentage of HPV-vaccinated female members aged 9–18 years—private health care (as of the end of 2021).

Number of Full HPV-Vaccinated (3 Doses) Female Members Aged 9–18 Years	Number of Female Members Aged 9–18	Percentage of Vaccinated Female Members Ages 9–18
1245	28,642	4.3%

**Table 9:** Number of participants in preventative actions from 2019 to 2021 (private health care).

	Year	2016	2017	2018	2019	2020	2021
Criterion							
Number of participants in preventative actions		No data available	No data available	No data available	265	104	700

**Table 10:** Proposed pattern of return to cervical cancer screening after interruption due to the COVID-19 pandemic.

Phase	Action	Evaluation Indicators
I	Resource Assessment Estimation of needs	Availability of personnel, materials, and equipment Number of people not screened by the deadline
II	Active telephone communication with unscreened individuals with appointments	Percentage of appointments with a scheduled test date
IIIa	Priority admission for those not tested	Percentage of outstanding screening tests performed
IIIb	Launch of screening for the general population	Percentage of screening coverage for the entire population

**Table 11:** Corrective action for the public cervical-cancer-screening program.

**Strategic Level**
✓ Acceptance of the vision for the screening program + updating current procedures and recommendations
✓ ✓ Justification of the viability of the project and securing funding
✓ Establishment of an annual goal and key performance indicators (KPIs)
**Management level**
✓ Ensuring an adequate number of medical personnel (adequate to the objectives set)
✓ Preparation of educational materials for medical staff and patients
✓ Planning the communication model
✓ Planning the IT architecture and data reporting model
✓ Periodic evaluation of performance and undertaking of corrective action
**Operational level**
✓ Medical staff and patients’ understanding of the proposed model and its benefits
✓ Prioritization among daily responsibilities and making time to complete assigned tasks
✓ Regular reporting of program progress and difficulties

**Table 12:** Plan for organizing HPV vaccination.

Plan for Organizing HPV Vaccination
✓ Introduction of a free national vaccination program
✓ Webinars on immunizations presented in schools, online, and on public TV
✓ Postgraduate education for physicians focused on the benefits of cervical cancer prevention
✓ Additional compensation based on the vaccination rate of the covered population
✓ Mobile vaccination points in rural areas
✓ Eligibility for vaccination by nurses (midwives) or pharmacists

## Data Availability

NHS and Private healthcare database—available at the request of the authors. Central Statistical Office (CSO) (https://stat.gov.pl/, accessed on 22 January 2022) and information on the functioning of the state telemedicine platform P1 (https://cez.gov.pl/; accessed on 22 January 2022).
